# Prophylactic Antibiotic Practices in Common Otologic Surgeries in Iran

**DOI:** 10.22038/IJORL.2021.56803.2959

**Published:** 2021-11

**Authors:** Mohammad Faramarzi, Ali Faramarzi, Sareh Roosta, Nikta Rabiei, Hossein Faramarzi

**Affiliations:** 1 *Department of Otorhinolaryngology Head and Neck Surgery, Shiraz University of Medical sciences, Shiraz, Iran.*; 2 *Otolaryngology Research Center, Shiraz University of Medical Sciences, Shiraz, Iran.*; 3 *Student Research Committee, Shiraz University of Medical Sciences, Shiraz, Iran.*; 4 *Department of Community Medicine, Faculty of Medicine, Shiraz University of Medical Sciences,* *Shiraz, Iran.*

**Keywords:** Antibiotics, Ear surgeries, Otologic surgeries, Prophylaxis

## Abstract

**Introduction:**

Rational surgical antibiotic prophylaxis is suggested for some selected surgical processes. However, inappropriate utilization of antimicrobial prophylaxis reduces benefits and increases costs and risks, such as antibiotic resistance. This study aimed to evaluate the current practice of antibiotics prescribed by surgeons in common otologic surgeries.

**Materials and Methods:**

This cross-sectional study was conducted among otolaryngologists with at least 5 years of experience in common otologic surgeries (tympanoplasty, tympanomastoidectomy, stapes, or middle ear exploration (MEE) surgeries). A total of 257 otolaryngologists filled a checklist about their selected regimen and timing of antibiotic(s) administration.

**Results:**

The rates of antibiotic prophylaxis prescription in dry and wet ears in tympanoplasty were 7.4% and 87.1% (preoperative), 40.9% and 47% (intraoperative), 88.3% and 98% (postoperative); in tympanomastoidectomy with no cholesteatoma were 7.1% and 97.8% (preoperative), 39.6% and 50.9% (intraoperative), 93.7% and 99.6% (postoperative); in tympanomastoidectomy with cholesteatoma were 14% and 98.3% (preoperative), 45.4% and 51.9% (intraoperative), 98.3% and 99.6% (postoperative), respectively, and in stapes or MEE surgeries were 6.4% (preoperative), 41.7% (intraoperative) and 73.1% (postoperative), respectively. There were no significant differences in the rates of prescribing intraoperative prophylaxis between wet and dry ears, except in tympanomastoidectomy without cholesteatoma. Overall, the most prescribed antibiotics were cephazolin, cephlexin, and ciprofloxacin drop.

**Conclusion:**

The results of this study revealed the inappropriate administration and timing of antibiotic prophylaxis regarding current literature evidence. Despite the lack of evidence on the potential role of antibiotic prophylaxis in clean-contaminated and contaminated ears, a significant number of surgeons prescribed prophylactic antibiotics in tympanoplasty and tympanomastoidectomy without cholesteatoma.

## Introduction

Surgical site infections (SSIs) are one of the main concerns in the field of medical surgery all around the world and account for 31% of all infections among hospitalized patients (1). Moreover, SSIs are the most common causes of nosocomial infection and the second most prevalent adverse event among hospitalized patients (2,3). Markedly, SSIs are the reasons for 77% of observed deaths in surgical patients. In this regard, it is estimated that 40-60% of infections can be prevented (2,3). This background provides the rationale of surgical antibiotic prophylaxis (SAP), albeit for some selected surgical processes (4,5).

There is evidence that the inappropriate utilization of SAP causes a reduction in its potential benefits and increases risks and costs (6). The results of some previous studies revealed the inappropriate administration of antimicrobial prophylaxis in various wards in Iran, such as the general surgical ward, the orthopedic ward (7), and the neurosurgery ward (8). The findings of a retrospective case study conducted by Khatami-Moghadam et al. also indicated the significant misuse of prophylactic antibiotics in general otolaryngologic surgeries in Iran (9). Preuss et al. in a survey on controversies in otology interviewed 100 otolaryngologists and found that 4% of the surgeons never used prophylactic antibiotics and 20% of them used antibiotics for non-infected ears (10). One of the significant disadvantages of SAP is that it increases the risk of antibiotic resistance (11,12). Based on the results of some studies, the excessive uses of antimicrobial medications and unsuitable time are still two common problems in surgical prophylaxis (13,14). Furthermore, the prophylaxis duration should not be prolonged postoperatively to reduce risks for antimicrobial resistance and other complications, such as Clostridium difficile diarrhea (15). Antibiotic therapy complications are expensive, with the estimated total costs of $1.5 billion annually for managing adverse antibiotic complications in the United States (16). In the light of this background, ensuring appropriate SAP in surgical wards and supporting its potential benefits should be a priority for surgeons. To the best of our knowledge, few studies have examined the use of prophylaxis antibiotics in ear surgeries. Therefore, this survey was conducted to illustrate the current status of using prophylaxis antibiotics in common otologic surgeries in Iran.

## Materials and Methods

This cross-sectional survey study was approved by the Research Ethics Committee of Shiraz University of Medical Sciences, Shiraz, Iran (IRB approval code: IR.SUMS.MED.REC.1397.486), and was conducted from December 2019 to January 2021. The primary inclusion criterion for otolaryngologists was having at least 5 years of experience in otologic surgery. Initially, a text message was sent to 650 otolaryngologists and asked them to participate in this voluntary survey. First, 488 specialists replied to our text message; however, 231 of them refused to cooperate, did not perform these surgeries, or did not have enough surgical experience, and consequently, were excluded from this study. Subsequently, invitations to complete the survey were sent to the otolaryngologists via text messages with no subsequent reminders. Finally, 257 otolaryngologists, who had at least 5 years of experience in these types of surgeries and met the inclusion criteria, were contacted either via phone or email to fill out the questionnaire anonymously and willingly.

The collected demographic data included the surgeons’ age, gender, and years of work experience. The questionnaires also consisted of questions on prescribing prophylactic antibiotics for seven specific types of surgeries, including tympanoplasty in dry and wet ears, tympanomastoidectomy in dry and wet ears with and without cholesteatoma, and stapes or middle ear exploration (MEE) surgeries, in pre-, intra-, and post-operative phases. If the antibiotics were prescribed, the properties of their selected regimen, either single or mixed therapy, the name of the antibiotic(s), and the timing of antibiotic(s) administration were mentioned regarding the specific type of operation.The phone conversation duration was about 3-30 min (median=5 min). The sample size was 237 persons, with the possibility of 90% for using antibiotics, a confidence level of 0.95, an error of 5%, and a design effect of 1.7 (n=Z*2pq/D*2).

## Results

Data were collected from 257 surgeons, including 216 (84%) males and 41 (16%) females. The mean scores of participants' age and work experience were 53.6±10.5 years (range 30-94) and 20.4±10.3 years (range 0.5-60). Concerning tympanoplasty in dry and otorrhea ears, 7.4% and 87.1% of the surgeons prescribed antibiotic prophylaxis 1-30 (median 2) and 1-42 (median 9.5) days before the operation, respectively. Moreover, 40.9% and 47% of the surgeons utilized antibiotics during the operation and 88.3% and 98% of them prescribed antibiotics 1-28 (median 7) and 1-20 (median 7) days postoperatively, respectively in dry and wet ears. The most prescribed antibiotic agents were cephazolin and Cipro drop (42.1% and 59.1% in dry and wet ears, respectively) in the preoperative phase, cephazolin (93.3% and 91.6% in dry and wet ears, respectively) in the intraoperative phase, and cephalexin (64.3% and 58.6% in dry and wet ears, respectively) after the operation. Regarding tympanomastoidectomy in dry and wet ears without cholesteatoma 7.1% and 97.8% of the surgeons prescribed antibiotic prophylaxis 1-30 (median 7) and 1-42 (median 10) days before the operation, 39.6% and 50.9% of the subjects used antibiotics during the operation, and 93.7% and 99.6% of the cases prescribed antibiotics 1-47 (median 7) and 1-20 (median 7) days after the operation, respectively. The most prescribed antibiotics were cephazolin and Cipro drop (38.9% and 48.9% in dry and wet ears surgeries, respectively) in the preoperative phase, cephazolin (93.1% and 88.7% in dry and wet ears surgeries, respectively) during the surgery, and cephalexin (63.2% and 56.4% in dry and wet ears surgeries, respectively) after the surgery. Considering tympanomastoidectomy among dry and wet ears with cholesteatoma, 14% and 98.3% of the surgeons prescribed antibiotic prophylaxis 1-30 (median 7) and 1-30 (median 8.5) days preoperatively, respectively. Also, 45.4% and 51.9% of the surgeons utilized antibiotics intraoperatively, and 98.3% and 99.6% of the surgeons prescribed antibiotics 1-47 (median 7) and 1-28 (median 7) days after surgery, respectively in dry and wet ears. The most prescribed agents in dry and otorrhea ear surgeries were Cipro drop (29.4% and 37.9% in dry and wet, respectively) before the surgery, cephazolin (92.7% and 85% in dry and wet, respectively) in the intraoperative phase, and cephalexin (56.7% and 53% in dry and wet, respectively) in the postoperative stage.There were no significant differences in the rate of intraoperative antibiotics prescription between wet and dry ears in either tympanoplasty (P=0.185) or tympanomas- toidectomy with cholesteatoma (P=0.158) surgeries; however, in tympanomastoidectomy without cholesteatoma operations, this rate was significantly higher in wet ears than in dry ones (P=0.013). Moreover, no significant differences were observed in the rate of prescribing antibiotic prophylaxis between tympanomastoidectomy with and without cholesteatoma in dry ears (P=0.187). In stapes or MEE surgeries, 6.4%, 41.7%, and 73.1% of the surgeons prescribed antibiotic prophylaxis in pre, intra, and postoperative phases, respectively. Additionally, the duration of antibiotic usage varied from 1 to 7 days before the operation (median=1.5) and 1 to 30 days after the operation (median=7). 

**Fig 1:**
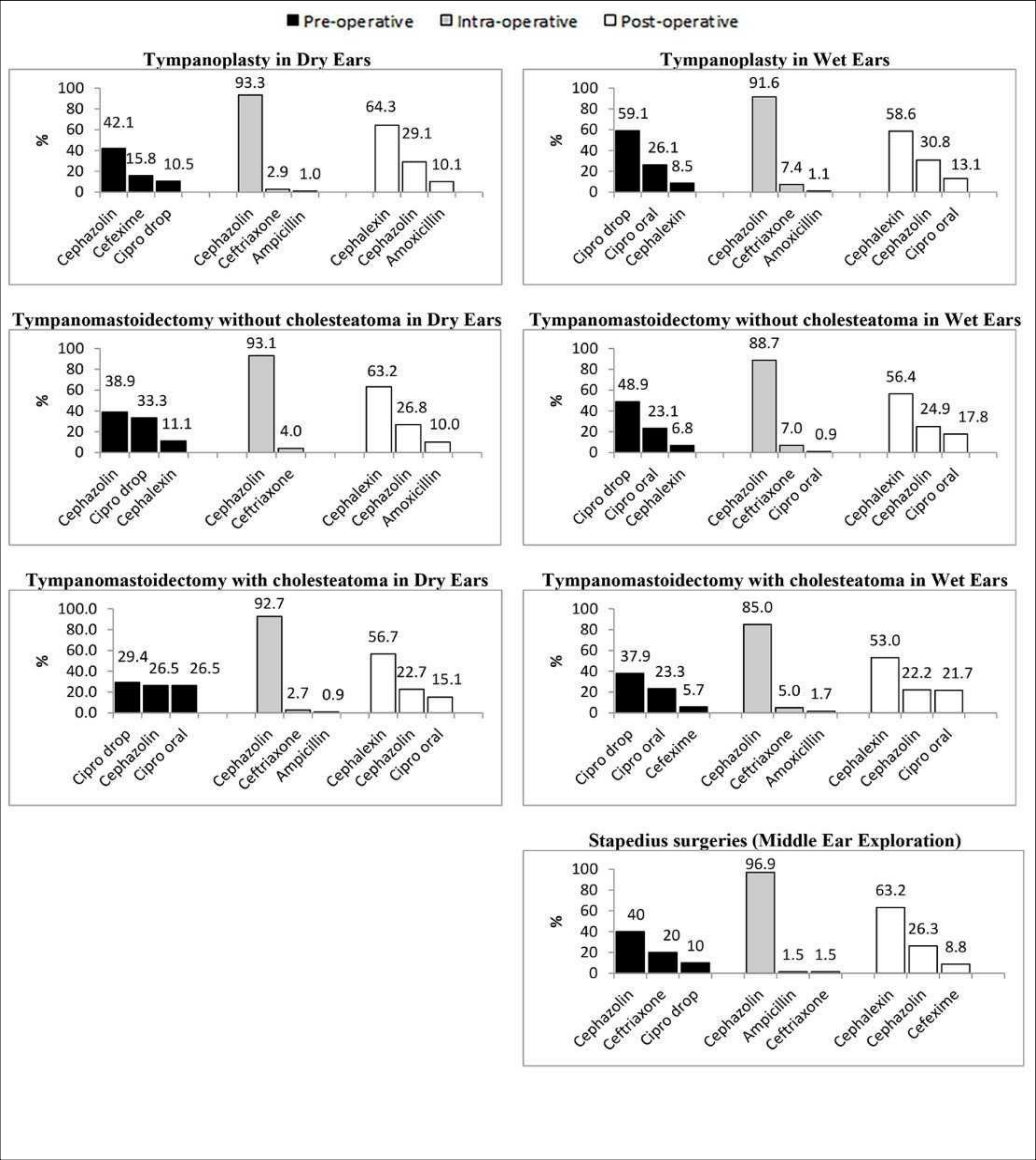
Most pre-, intra-, and post-operatively prescribed antibiotic prophylaxis in different operations

Furthermore, the most pre-, intra-, and post-operatively prescribed antibiotics were cephazolin (40%), cephazolin (96.9%), and cephalexin (63.2%), respectively (Table 1) (Figure 1).

**Table 1:** Using antibiotic prophylaxis by surgeons in different operations

Tympanoplasty in dry (n=257) and wet (n=202) ears						
Antibiotic Usage	Pre-operative		Intra-operative		Post-operative	
	Dry	Wet	Dry	Wet	Dry	Wet
**n (%)**	19 (7.4)	176 (87.1)	105 (40.9)	95 (47.0)	227 (88.3)	198 (98.0)
**Days**	5.2±9.5(1-30, 2)^a^	10.6±5.8(1-42, 9.5)			7.0±3.0(1-28, 7)	7.5±3.0(1-20, 7)
Tympanomastoidectomy in dry (n=255) and wet (n=226) ears with no cholesteatoma						
**Antibiotic Usage**	Pre-operative		Intra-operative		Post-operative	
	Dry	Wet	Dry	Wet	Dry	Wet
**n (%)**	18 (7.1)	221 (97.8)	101 (39.6)	115 (50.9)	239 (93.7)	225 (99.6)
**Days**	8.3±8.6(1-30, 7)	10.3±5.4(1-42, 10)			7.6±4.0(1-47,7)	7.6±2.8(1-20, 7)
Tympanomastoidectomy in dry (n=242) and wet (n=231) ears with cholesteatoma						
**Antibiotic Usage**	Pre-operative		Intra-operative		Post-operative	
	Dry	Wet	Dry	Wet	Dry	Wet
**n (%)**	34 (14.0)	227 (98.3)	110 (45.4)	120 (51.9)	238 (98.3)	230 (99.6)
**Days**	9.3±6.9(1-30, 7)	9.8±5.3(1-30, 8.5)			8.1±4.0(1-47, 7)	8.2±3.2(1-28, 7)
Stapes or MEE surgeries (n=156)						
**Antibiotic Usage**	Pre-operative		Intra-operative		Post-operative	
**n (%)**	10 (6.4)		65 (41.7)		114 (73.1)	
**Days**	2.75±2.9(1-7, 1.5)				6.9±4.1(1-30, 7)	

## Discussion

The present study was designed to determine the otolaryngologists' tendency to prescribe antibiotics in common otologic surgeries. The results indicated that in wet ear operations, most of the surgeons prescribed preoperative antibiotics, and approximately half of them prescribed intraoperative antibiotics. Moreover, the majority of the surgeons administered postoperative antibiotics in both dry and wet ear surgeries. The findings of the current study showed that seemingly antibiotic prophylaxis needed to be more evidence-based. 

A review of the relevant literature has indicated that the use of SAP in clinical practices is inadequately highlighted in terms of administration time of the first dose, choice of antibiotic, lack of intraoperative dose administration, and prolonged use of antibiotics (17). According to four surgical wound categories specified by the Center for Disease Control, the surgical wounds are classified as clean-contaminated in dry ears and stapes or MEE surgeries, contaminated in wet ear operations, and dirty or infected in ears with cholesteatoma (18). Little evidence is available on the potential role of antibiotic prophylaxis in contaminated ear cases (19). For example, Zhang et al. in their meta-analysis revealed the safety and efficacy of antibiotic prophylaxis in clean high-risk and clean-contaminated plastic surgeries (4). On the contrary, some pieces of evidence show that preoperative antibiotic prophylaxis confers no benefit in reducing the infection rate of mandibular fractures and is regularly used in maxillofacial trauma practices (5). Moreover, Preuss et al. found that 56% and 48% of surgeons gave perioperatively a single-shot and preoperatively antibiotic in infected ears (10). The findings of the present study showed that 40.9% of the surgeons prescribed antibiotics intraoperatively in dry ear tympanoplasty; however, 52.97% of the cases did not prescribe antibiotics in wet ear tympanoplasty. Consequently, it appears that the current practice needs to be supported by further evidence. Non-evidence-based prophylactic antibiotic prescription is one of the global problems, which is more remarkable in developing countries (20). Furthermore, the findings of previous studies conducted in Iran are indicative of the antibiotic overuse problem (21,22,8).

Regarding the administration rate of postoperative antibiotics and the type of surgery, the results of the present study showed that the surgeons' antibiotic prescription rates in postoperative tympanoplasty were 88.3% and 98.02% in dry and wet ears, respectively. In comparison, Raine et al. reported this rate as 59% in their survey in the United Kingdom (23). The literature abounds with studies that either reject or advocate prophylactic antibiotic prescription in tympanoplasty (23-27). In their study, Govaerts et al. concluded that dry perforation tympanoplasties needed to be considered as clean operations based on the American National Research Council, although they lacked the advantages of antibiotic prophylaxis (28). Moreover, the prophylactic antibiotic prescription rate in tympanomas- toidectomy with cholesteatoma was 99.6% in wet ears. In a retrospective review by Pierce et al., patients cured without and with antibiotics had surgical site infection rates of 11% and 1% (P=0.02), respectively. In conclusion, they found that the administration of antibiotics may prevent SSIs in this type of surgery (29). In our study, the prophylactic antibiotic prescription rates in stapes surgeries were 6.4% (preoperative), 41.7% (intraoperative), and 73.08% (postoperative). However, the results of a systematic review by Patel et al. on the evidence-based use of antibiotics for common otolaryngology operations did not support routine antibiotic prophylaxis for this type of surgery (26).

As Table 1 presents, the median duration of post-operative prophylaxis prescription in this survey was 7 days. Some studies, such as a systematic review and meta-analysis performed by Oppelaar et al. (30) and another review study conducted by Patel et al. (26), evaluated prolonged and short courses of antibiotic prophylaxis following otorhinolaryngology surgeries. They found no difference in the occurrence of post-operative infections between short-course (24-48) and extended-course, implying that the short-course is preferred (26,30). 

 In terms of the type of antibiotic selected by surgeons, our findings showed that ciprofloxacin otic drop and cephazolin in pre-operative, cephazolin in intra-operative, and cephalexin in the postoperative stage were the most prescribed antibiotics. In addition, the results of an Iranian single-centered retrospective chart review study indicated that the main pre-operative prescribed antibiotics were cephalosporins in general otolaryngology surgeries (9). Since there are neither local nor international guidelines for otolaryngology surgeries, a wide range of variations in the type of prescribed antibiotics is observable in literature (26). A Cochrane review on 11 randomized controlled trials evaluating the effectiveness of antibiotic prophylaxis in-ear surgeries found no difference in postoperative infection and graft success rate when comparing placebo, intra-operative usage, and prolonged postoperative usage (31). The findings of the mentioned study indicated that there was no adherence in terms of the type of prescribed antibiotics; however, cephalosporins and ampicillin relatively had more prescription rates (31).

Surprisingly, there was no significant difference in the rate of intra-operative prophylactic antibiotic prescription between wet and dry ears in both tympanoplasty and tympanomastoidectomy with cholesteatoma groups. In spite of surgical wounds in ears with cholesteatoma classified as dirty or infected and in other ears classified as clean-contaminated or contaminated (18), remarkably our findings showed no significant difference in the rate of prescribing antibiotic prophylaxis between tympanomastoidectomy with and without cholesteatoma in both wet and dry ears.

## Conclusion

The study findings revealed a considerable variation of antibiotic prophylaxis prescription, duration, and type in common otologic surgeries in comparison with literature evidence. One of the mainstays of appropriate prophylaxis antibiotics is to change the healthcare system from the push by cultural and institutional routines for adherence to a patient-centered base. Regarding current literature evidence, the results of this study showed inappropriate administration and timing of antibiotic prophylaxis. To provide a rationale based on the concepts of antibiotic prophylaxis efficiency in common otologic surgeries, it is recommended to develop local and international guidelines. Regarding the improved quality in surgical wards, surgeons should prevent the misuse of antibiotic prophylaxis by paying attention to the Hippocratic maxim of “do not harm patients.”
